# Integrated Proteomics Based on 2D Gel Electrophoresis and Mass Spectrometry with Validations: Identification of a Biomarker Compendium for Oral Submucous Fibrosis—An Indian Study

**DOI:** 10.3390/jpm12020208

**Published:** 2022-02-03

**Authors:** Divyambika Catakapatri Venugopal, Soundharya Ravindran, Vidyarani Shyamsundar, Sathasivasubramanian Sankarapandian, Arvind Krishnamurthy, Ananthi Sivagnanam, Yasasve Madhavan, Vijayalakshmi Ramshankar

**Affiliations:** 1 Department of Oral Medicine and Radiology, Sri Ramachandra Institute of Higher Education and Research (DU), Porur, Chennai 600116, India; cvdivyambika@sriramachandra.edu.in (D.C.V.); dr_sathasivam@yahoo.co.in (S.S.); yasasvem@gmail.com (Y.M.); 2 Department of Preventive Oncology (Research), Cancer Institute (WIA), Adyar, Chennai 600020, India; soundharyaravindran@gmail.com (S.R.); balaananthi20@gmail.com (A.S.); 3 Centre for Oral Cancer Prevention and Research, Sree Balaji Dental College and Hospital, Pallikaranai, Chennai 600100, India; drvidyaranishyam78@gmail.com; 4 Department of Surgical Oncology, Cancer Institute (WIA), Adyar, Chennai 600020, India; drarvindkrishnamurthy@gmail.com

**Keywords:** OSMF, proteomics, MALDI, immunohistochemistry, gene ontology

## Abstract

Oral Submucous Fibrosis (OSMF) is a chronic debilitating disease more frequently found in the South East Asian population. This disease poses a public health priority, as it is grouped under oral potentially malignant disorders, with malignant transformation rates of around 7 to 13%. Hence, early identification of high-risk OSMF patients is of the utmost importance to prevent malignant transformation. Proteomic expression profiling is a promising method for identifying differentially expressed proteins for disease prognosis and risk stratification in OSMF. In this study, overexpressed proteins in OSMF, OSMF transformed into oral squamous cell carcinoma (OSCC) and normal tissues were evaluated by proteomic analysis using two-dimensional electrophoresis (2DE) and mass spectrometry, which revealed 23 upregulated proteins. Validation was done using immunohistochemistry for three secretory proteins, namely 14-3-3ε (*n* = 130), carbonic anhydrase 1 (CA 1) (*n* = 125) and heat shock protein 70 (HSP 70) (*n* = 117), which showed significant overexpression in OSMF, OSCC compared to normal. The present study is the first of its kind in India to the best of our knowledge, assessing the altered expression of proteins in OSMF and OSMF which has undergone malignant transformation, obtaining a better knowledge of the molecular pathways involved in the disease progression. The current study shows that the biomarkers studied can be potentially useful for risk stratification of OSMF to OSCC serving as novel targets for therapeutic intervention. Clinical validation of the targets can further pave way for precision medicine to improve the quality of life in OSMF patients.

## 1. Introduction

Oral submucous fibrosis is a chronic, potentially malignant disorder of the oral mucosa, which is more widespread in the Indian subcontinent [1,2]. The main etiological determinant is the areca nut, which stimulates fibroblast proliferation and collagen synthesis and also reduces collagen breakdown. It consists of alkaloids, namely arecoline, arecaidine, guvacine and guvacoline, which undergo nitrosation to form nitrosamines, which further alkylates with DNA, leading to malignant transformation on prolonged exposure. Listed as a Group I carcinogen by the International Agency for Research on Cancer, the areca nut is a common component of betel quid, which is predominantly used in Southeast Asia [3]. Among the various oral potentially malignant disorders (OPMD), OSMF has a significant malignant transformation rate, ranging from 7 to 13% [4]. Chewing areca nut causes continuous local irritation, leading to injury-related chronic inflammation, oxidative stress, and cytokine production. Oxidative stress and the successive creation of reactive oxygen species (ROS) induce cell proliferation, cell aging or apoptosis, depending on the amount of ROS production. In the event of chronic exposure, these events lead to preneoplastic changes in the oral cavity and, subsequently, to oral malignancy [5]. Furthermore, the epithelial to mesenchymal transition has been found to be involved in the pathogenesis of OSMF. The betel quid induced tissue injury releases ROS, mediating TGF β-induced epithelial to mesenchymal transition and playing an important role in the fibrosis of OSMF [6].

Studies on proteomic analysis in OSMF have been found to be sparse in the existing literature. The earlier study performed in oral cancers progressing from OSMF showed that ANXA4 and FLNA have great prognostic value for patient survival, which could be potential targets for therapeutic interventions [7]. In another study, two-dimensional electrophoresis-based proteomic approaches were used to detect the differentially expressed proteins between OSMF and normal tissue. A total of 88 proteins with altered expression levels were identified, and cyclophilin A was proposed as a potential biomarker and therapeutic target for OSMF [8]. In addition, a study conducted in India showed differentially expressed proteins between OSMF and normal tissue by a proteome analysis with two-dimensional electrophoresis and Matrix-Assisted Laser Desorption Ionization Imaging Time of Flight (MALDI TOF) mass spectrometry, which showed that 15 proteins were upregulated and that 10 proteins were downregulated in the OSMF tissues compared to normal tissue [9]. MALDI-IMS-based proteome analysis, used to analyse the differences in protein expression between OSCC tissues and adjacent non-cancerous OSMF tissues, showed nine differently expressed proteins, of which the expression of NCOA7 in OSCC tissues was upregulated by immunohistochemical staining and Western blotting and correlated with the clinicopathological parameters [10].

With the current existing knowledge, studies concerning proteomic profiling in OSMF are sparse. The present study shows the proteomic expression profile in normal, OSMF and OSCC samples, with validation of the top upregulated proteins using immunohistochemistry (IHC) for a better understanding of protein targets. Our study shows comprehensive differentially expressed profiles of OSMF and OSMF along with OSCC in the same sample compared with the normal samples. The targets have been additionally validated in a large series of clinical samples.

## 2. Materials and Methods

### 2.1. Patient Tissue Samples

The study was approved by the Institutional Ethical Committee (IEC No. 19/DEC/72/118) and was conducted at the Department of Oral Medicine and Radiology, Sri Ramachandra Institute of Higher Education and Research, from December 2019 until January 2021. Written informed consent was obtained from all participants, followed by the collection of tissue samples. Patient demographic details, medical history, habits and details of clinical examination were recorded in the proforma. Patients with no habit of chewing areca nut and no clinical signs of OSMF were included in the normal group, and mucosal tissue was collected during the extraction of the third molar. Patients who were clinically diagnosed with OSMF with histological confirmation and patients who were clinically and histologically diagnosed with OSCC along with pre-existing OSMF were included in their respective study groups. In addition, formalin-fixed paraffin-embedded (FFPE) sections from normal, OSMF and OSCC were obtained for validation studies. All the tissue samples were snap-frozen and stored in liquid nitrogen until used for RNA and protein extraction.

### 2.2. Protein Extraction from Tissue Samples

Tissue extracts were made by crushing the pooled samples (Normal = 10, OSMF = 12, OSMF with OSCC = 6) with liquid nitrogen in a pre-chilled mortar-pestle, and they were then dissolved in a lysis buffer (7M CO(NH₂)₂, 2M SC(NH₂)₂, 4% CHAPS, 20mM phenylmethylsulfonyl fluoride and 20mM dithiothreitol). Tissue samples were then sonicated for 10 min and centrifuged for 15 min at 4 °C at 12,000 rpm [11]. The proteins extracted were estimated using the Bradford method before being aliquoted and stored at −80 °C for further analysis.

### 2.3. 2D Gel Electrophoresis

Two-dimensional electrophoresis of the treated proteins was performed as previously stated [12]. In the first dimension, 13cm IPG strips of pH 3-10 (GE Healthcare, Uppsala, Sweden) were used, and an active/passive rehydration process was carried out. Proteins were focused for 50,000 Vhs in an IPGPhor III (GE Healthcare, Uppsala, Sweden) apparatus with the following IEF conditions: 100 V gradient for 1 h, 300 V gradient for 2 h, 1000 V gradient for 1 h, 5000 V gradient for 5 h, and 5000 V step and held for 7 h at a constant temperature (20 °C). Following isoelectric focusing (IEF), each IPG strip was placed in an equilibration solution containing 2% DTT, followed by incubation in another buffer containing 2.5% iodoacetamide in place of DTT. The second dimension PAGE (12.5%) was performed in an SE600 (GE Healthcare, Uppsala, Sweden) at 1W/gel for 1 h and 13W/gel for 3 h.

### 2.4. In-Gel Trypsin Digestion and MALDI-TOF

Proteins were stained separately with colloidal Coomassie blue G-250 and scanned with a high precision scanner (ScanMaker 9700XL, Microtek). The gel image analysis programme PDQuest 8.01 (Bio-Rad) was used to detect protein expression levels [13]. In the gel, the protein spots of interest were digested with trypsin before being examined by mass spectrometry. Gel fragments were rinsed with Milli-Q water before being treated with a decolorizing solution containing 50% acetonitrile and 25% ammonium bicarbonate. Discoloured gel fragments were thoroughly dehydrated in 100% acetonitrile (ACN) for 10 min before being vacuum dried for 30 min.

The gel pieces were rehydrated/trypsinized for 30 min on ice in 5 litres of trypsin buffer (10 mM ammonium bicarbonate in 10% ACN) containing 400 ng trypsin (Sigma Aldrich, USA) and then incubated for 16 h at 37 °C in 25 litres of buffer (40 mM ammonium bicarbonate in 10% ACN). Following incubation, the peptides were extracted twice by sonication (10 min) with 25 litres of 0.1% trifluoroacetic acid (TFA) in 60% ACN, followed by 20 litres of 100% ACN. Extracted peptides were vacuum-dried for 90 min and kept at 4 °C [14,15]. Mass accuracy was externally calibrated for peptide mass fingerprint analysis using a peptide standard range of 700–4000 Da. Internal calibration was performed using enzyme autolysis peaks. The raw spectra produced by MS were analysed using the SNAP algorithm in the FlexAnalysis software 2.4 (Bruker Daltonics). Searching the NCBI database with the Mascot search engine V2.2 (Matrix Science, UK) was performed [16,17].

### 2.5. Pathway Analysis and Gene Ontology

The gene symbols of differentially expressed proteins were added into the PANTHER (www.pantherdb.org (accessed on 8 December 2021)) database for functional categorization and pathway analysis. STRING (www.string.db.org (accessed on 8 December 2021)) was used to create protein networks [18]. Correlations were formed directly (physically) and indirectly (functionally) from four separate sources: genetic context, high throughput testing, prior knowledge, and conserved co-expression. The integration maps were built utilizing quantitatively combined interaction data from multiple sources.

### 2.6. Immunohistochemistry

Immunohistochemistry was done on 4µm obtained from formalin-fixed paraffin-embedded tissue (FFPE) samples. Sections were taken on slides coated with 3-aminopropyltriethoxysilane (APES). The sections were deparaffinised in xylene and rehydrated using absolute alcohol. Endogenous peroxidase activity was quenched by immersing the sections for 10 min in 0.03% hydrogen peroxide in distilled water, followed by a distilled water wash. Antigen retrieval was done with 0.05M Tris EDTA Buffer (pH-9) in a pressure cooker for 20 min. Sections were pre-incubated with 2% bovine serum albumin (BSA) for 40 min. The sections were incubated with primary antibody CA 1 (Santa Cruz-393490 CA1 antibody F-5; 1:50 dilutions in 1% BSA), 14-3-3ε (Santa Cruz-23957 14-3-3ε antibody 8C3; 1:250 dilutions in 1% BSA) and HSP 70 (Vitro SA-MAD000531Q mouse anti-human HSP 70 monoclonal antibody clone W27; prediluted) overnight at 4 °C in 100% moisture. The Polyexcel HRP/DAB detection system (PathnSitu, 1257 Pleasanton, CA, USA) was used to detect expression. Hematoxylin counterstained sections were dehydrated using ascending grades of isopropyl alcohol and xylene and mounted in DPX. Known positive controls and negative controls were used. The expression of 14-3-3ε, CA 1 and HSP 70 was graded and compared to the absolute normal oral mucosa. In 10 high power fields (40X), several positive cells were counted in the epithelium and connective tissue (connective tissue cells like fibroblasts and inflammatory cells), and the % positivity was computed. Counting was done on a computer display using the software ProgRes CapturePro v2.8.8 [19,20,21]. Briefly, CA 1, HSP 70 and 14-3-3ε expression was assessed semi-quantitatively by evaluating the percentage of epithelial and CT cells, 20% or more, expressing the respective proteins considered positive. The staining intensity was measured at several levels of the epithelium (basal, stratum spinosum and superficial). Similarly, expression in the connective tissue was also counted. Scoring for IHC was done by an oral pathologist who was blinded to the clinical details of the included samples [22,23,24].

### 2.7. Statistical Analysis

The student’s *t*-test was employed in 2D-gel electrophoresis to calculate statistically significant differences in the relative abundance of particular protein spots between two groups. A value of *p* < 0.05 was considered statistically significant. Clinicopathological parameters and immunoexpression-based correlations were done using SPSS (IBM Corporation version 16) [25].

## 3. Results

### 3.1. Quantitative Protein Profiling Using 2D Gel Electrophoresis and MALDI-TOF

**Figure 1:**
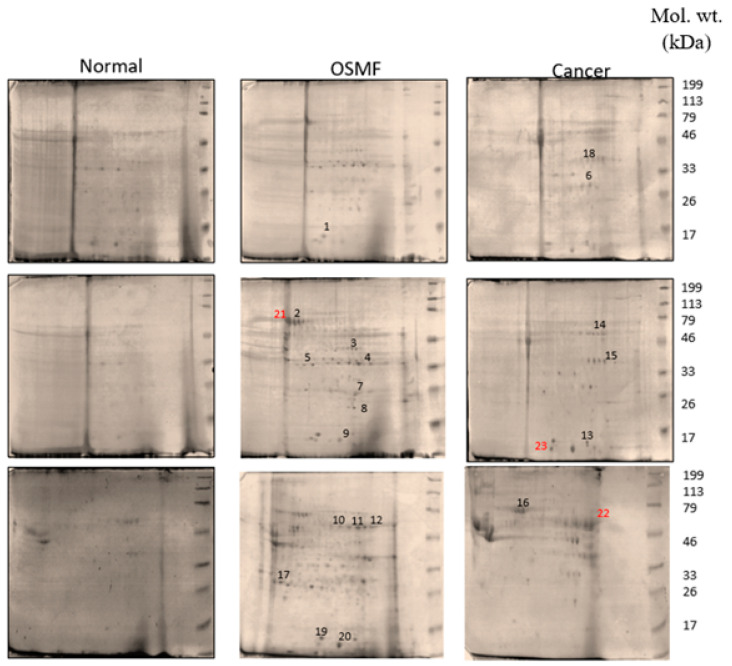
Representative 2DE gel images of normal, OSMF and OSCC tissue samples.

A comparative proteomic study of pooled tissue samples was taken from patients with OSMF, OSMF with histologically proven OSCC and was compared to pooled normal samples (Figure 1). The image analysis platform yielded more than 50 protein pairs, 23 of which were upregulated in tumour samples when compared to nearby normal protein samples. The top 23 differentially expressed proteins were chosen for mass spectrometry from the list of differentially expressed proteins. Table 1 shows the protein identification details for the differentially regulated proteins, including the database accession number, mass spectroscopy probability score and percentage of sequence coverage match. The top 23 differentially upregulated proteins are listed in Table 2, in which three secretory proteins, namely carbonic anhydrase 1 (CA 1), 14-3-3 epsilon (14-3-3ε) and heat shock protein 70 (HSP 70), were taken up further in the current study for validation with immunohistochemistry.

### 3.2. Functional Classification of Identified Proteins and Biological Network Analysis

**Figure 2:**
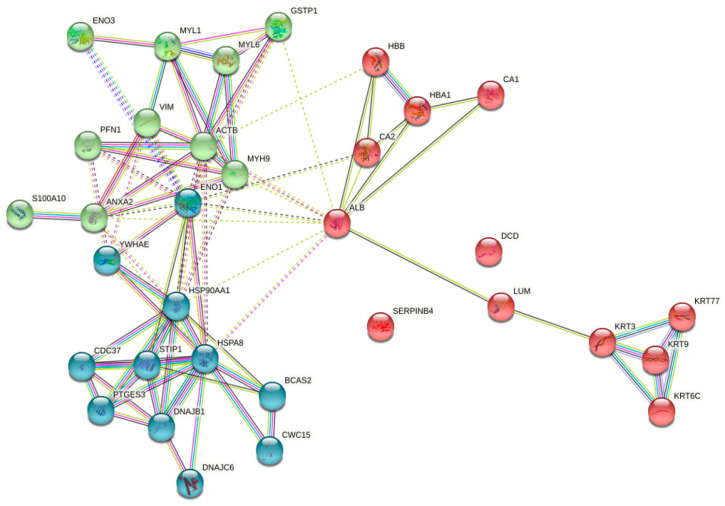
Representative 2DE gel images of normal, OSMF and OSCC tissue samples.

The STRING database was used to analyse protein–protein interactions for all 23 differentially elevated proteins. A cluster analysis found that among the 23 proteins discovered, two (Serpin B4 and Dermcidin) stood out as individual actors and had not been documented to interact with other proteins identified in this study. The remaining proteins interacted with one another, either directly or indirectly, via other protein networks (Figure 2). Serum albumin interacted with alpha-enolase, heat shock protein, myosin and lumican to form a network that connected to other protein networks such as keratin and annexin proteins. A cluster analysis was performed for all the interaction networks by K means clustering, and they were grouped into three major clusters; one was with all contractile proteins, conserved proteins (HSP), cytosolic proteins (carbonic anhydrase, enolase) including profilin, myosin light chain family members and tropomyosin. Other major clusters included all heat shock proteins, inflammatory proteins, serpin family proteins and annexin.

**Figure 3:**
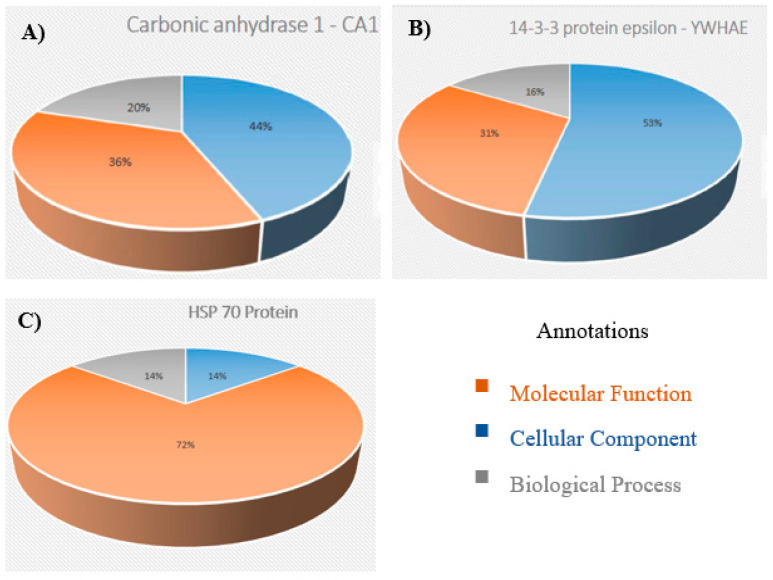
Functional classification of (**A**) CA 1, (**B**) 14-3-3ε and (**C**) HSP 70 using the PANTHER gene ontology database.

All differently expressed upregulated proteins were divided into three groups based on their molecular function, their biological process and their cellular components (Figure S1A,C). For the molecular function of the identified proteins, most of them had binding and catalytic activity, followed by transporter activity. Most of them were cytoskeletal proteins and were mainly involved in the cellular and metabolic processes (Figure S2A). When all functional categories were summarized, it was very obvious that all these differently expressed proteins played a critical role in tumour development, as they were mainly involved in biological regulation, including glycolysis, the epidermal growth factor/fibroblast growth factor signalling pathway, the cytoskeletal system regulation by Rho-GTPase and the apoptosis signalling pathway (Figure S2B). It was found that the three differently expressed secretory proteins (CA 1, 14-3-3ε and HSP 70), which were considered for further validation, were mainly involved in cellular processes, biological regulation and molecular functions (Figure 3 and Tables S1–S3).

### 3.3. Validation Studies in Clinical Samples Using Immunohistochemistry

Immunohistochemistry was done using clinical samples of normal, OSMF and OSCC. Initially, IHC was performed with a sample size of *n* = 130 for the three proteins, namely CA 1, 14-3-3ε and HSP 70. However, five sections of CA 1 and 13 sections of HSP 70 got washed off during the IHC procedure and hence were not available for scoring and statistical analysis. Thus, the final sample size taken for statistical evaluation was *n* = 130 for 14-3-3ε, *n* = 125 for CA 1 and *n* = 117 for HSP 70.

**Figure 4:**
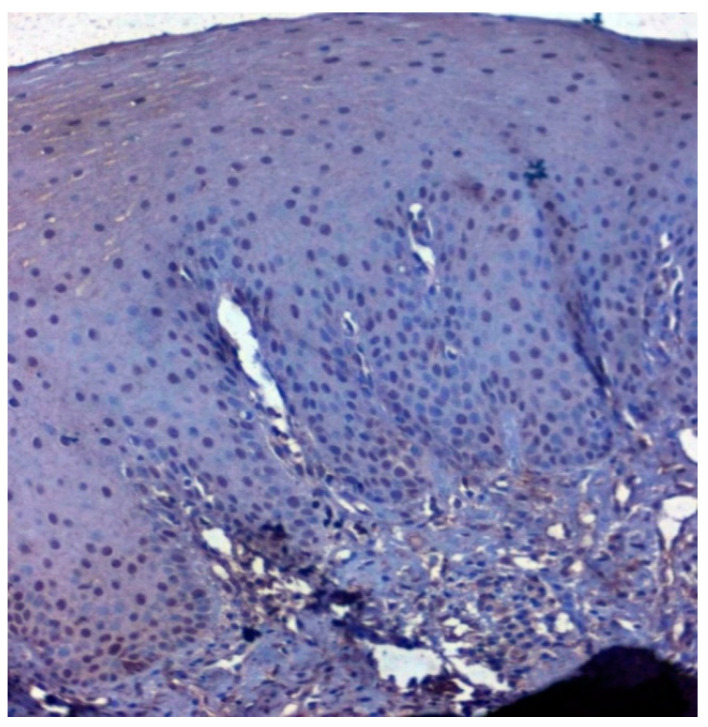
IHC for CA 1 in normal samples (under 20× magnification).

**Figure 5:**
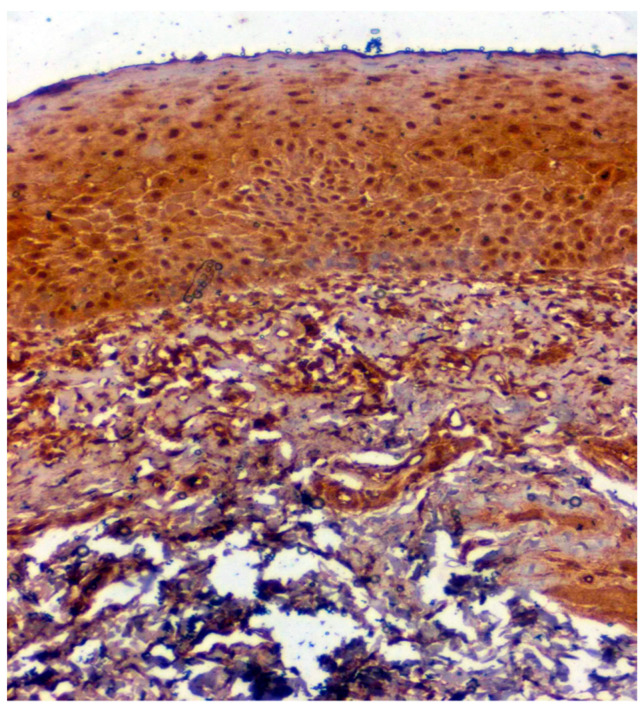
IHC of CA 1 in OSMF samples (under 20× magnification).

**Figure 6:**
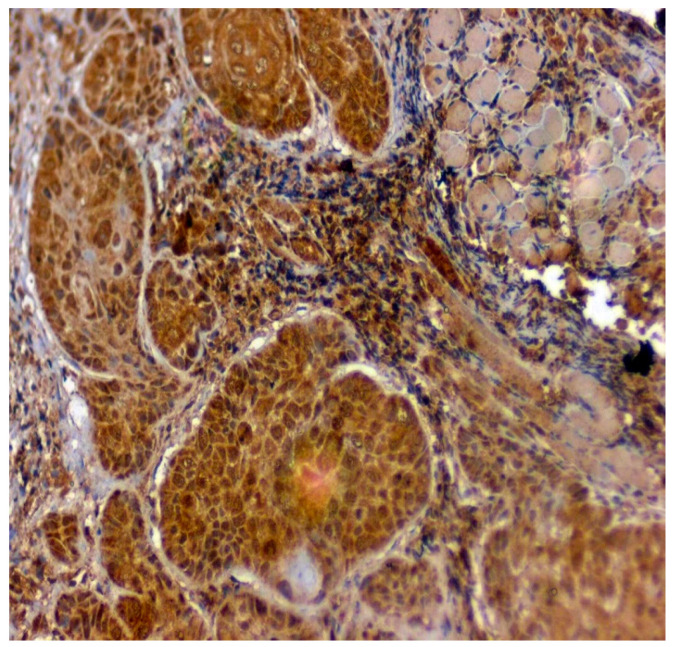
IHC for CA1 in OSCC samples (under 20× magnification).

Positive immunoexpression for CA 1 was found in 70.4% (88/125). Among the 40 patients with OSCC, CA 1 overexpression was found in 75% (30/40), and among the OSMF patients, CA 1 overexpression was found in 77.8% (56/72) compared to 15.4% (2/13) of normal, which was found to be statistically significant (*p* = 0.000; χ2 = 21.169) (Table 3). Based on different degrees of epithelial abnormalities, we found a statistically significant association (*p* = 0.025; χ2 = 11.144). Our results showed epithelial atrophy among the OSMF patients showing positive CA 1 overexpression. There was no significant association between CA 1 expression and degrees of inflammation, fibrosis and vascularity. Figure 4 shows the negative immunoexpression of CA1 in normal samples. Figure 5 shows the CA 1-positive immunoexpression in epithelial cells in OSMF samples, with cytoplasmic and nuclear positivity. In OSCC samples, CA 1 demonstrates strong cytoplasmic and nuclear positivity in malignant epithelial cells (Figure 6).

**Figure 7:**
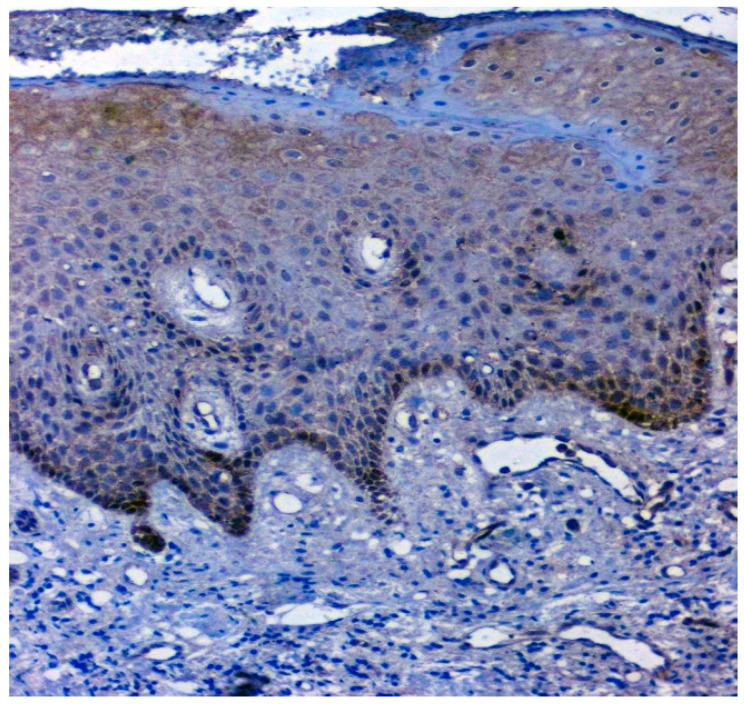
IHC for 14-3-3ε in normal samples (under 20× magnification).

**Figure 8:**
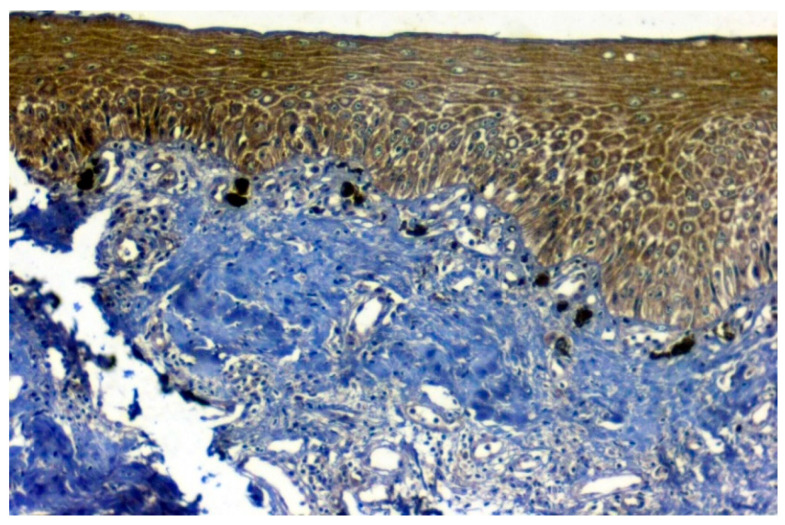
IHC for 14-3-3ε in OSMF samples (under 20× magnification).

**Figure 9:**
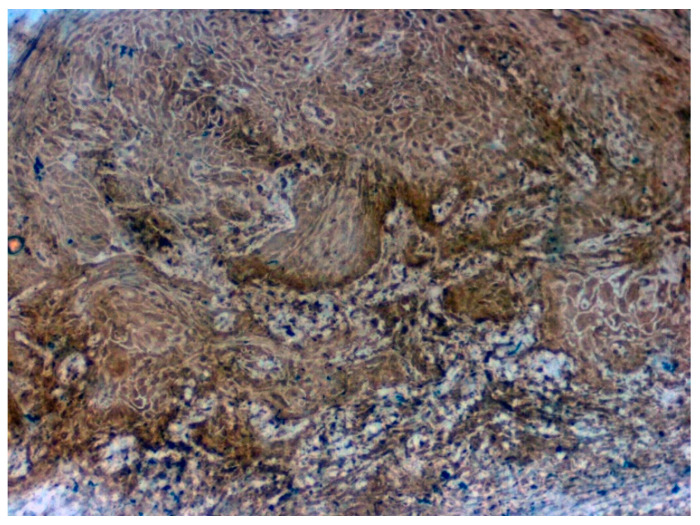
IHC for 14-3-3ε in OSCC samples (under 20× magnification).

The expression of 14-3-3ε was studied in 130 patients, of which 81.5% (106/130) were found to be expressing 14-3-3ε. There was a significant correlation (*p* = 0.000; χ2 = 33.600) with expression among normal vs. OSMF vs. OSMF vs. OSCC patients. Table 4 shows the details of 14-3-3ε expression, which correlated with clinicopathological parameters. We found a statistically significant overexpression (*p* = 0.019; χ2 = 5.496) with increased age (>43 years), atrophic epithelium (*p* = 0.011; χ2 = 13.149) and increased degrees of inflammation (*p* = 0.041; χ2 = 8.252). Figure 7 shows the negative immunoexpression of 14-3-3ε in normal samples. Figure 8 shows the 14-3-3ε-positive immunoexpression in epithelial cells in OSMF samples, with cytoplasmic and nuclear positivity. In OSCC samples, 14-3-3ε demonstrates strong cytoplasmic and nuclear positivity in malignant epithelial cells (Figure 9).

**Figure 10:**
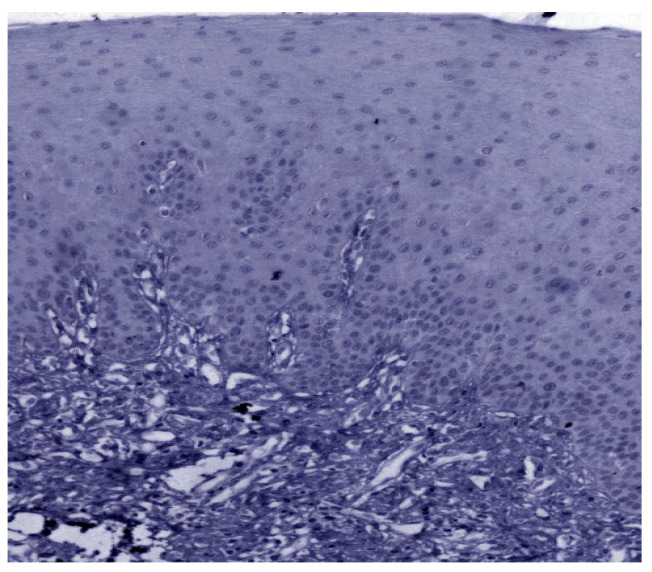
IHC for HSP 70 in normal samples (under 20× magnification).

**Figure 11:**
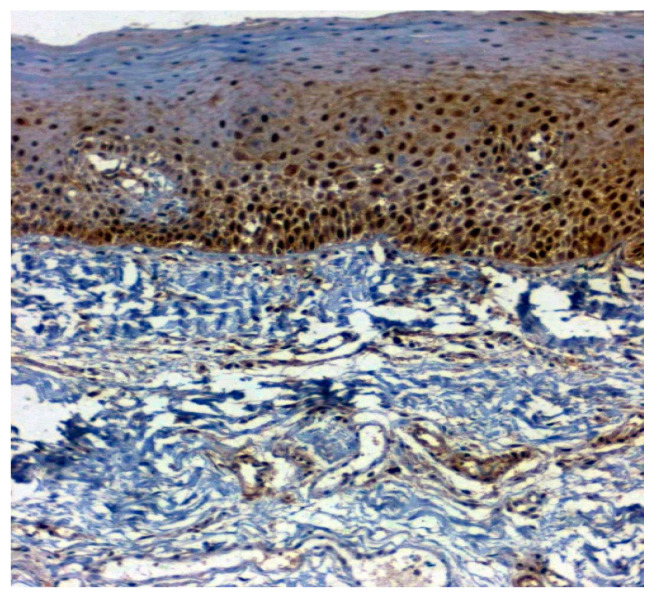
IHC for HSP 70 in OSMF samples (under 20× magnification).

**Figure 12:**
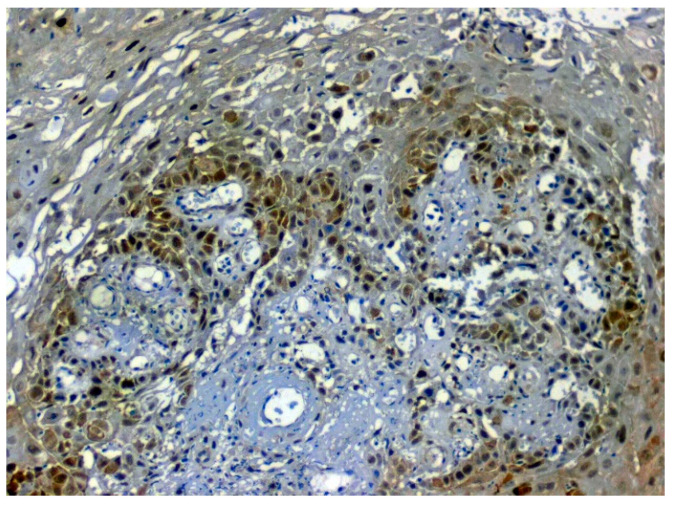
IHC for HSP 70 in OSCC samples (under 20× magnification).

The expression of HSP 70 was studied in 117 patients, of which positive immunoexpression was found in 69.2% (81/117) patients. HSP overexpression was increased in OSCC oral cancer and OSMF compared to normal (*p* = 0.012; χ2 = 8.805). HSP overexpression significantly correlated (*p* = 0.016; χ2 = 6.705) with increased age (>47 years). HSP overexpression also showed a significant correlation with epithelial abnormality with increasing grades of dedifferentiation (*p* = 0.05; χ2 = 9.313) (Table 5). Figure 10 shows the expression of HSP 70 in normal oral samples showing negative epithelial cells. Figure 11 shows an expression of HSP 70 in OSMF samples showing intense cytoplasmic and nuclear positivity. Figure 12 shows the expression of HSP 70 in OSCC samples showing intense cytoplasmic and nuclear positivity.

## 4. Discussion

The current study shows CA 1, 14-3-3ε and HSP 70 to be potentially useful markers in identifying OSMF patients who are at the risk of malignant transformation and require immediate intervention. Risk stratification of OPMD requires diagnostic tools with increased specificity and sensitivity, which will enable early detection of oral cancer [26]. The current study has shown that the discovery of protein biomarkers based on proteomic analysis can help in the assessment of disease prognosis and also aid in the development of targeted therapy, as described in existing literature [27,28]. We identified 23 top upregulated proteins in OSMF and validated proteins, namely 14-3-3ε, CA 1 and HSP 70. Previous studies have shown that proteomic analysis employs better efficiency approaches supported by bioinformatics to identify and quantify the total protein content of cells, tissues or biological fluids [29]. Proteomic analysis helps in assessing the protein interactions involved in the cellular process, thereby aiding in understanding the molecular pathogenesis of the disease [30]. MALDI-TOF, which was employed in our study, is known for its sensitivity, ease of use and its capacity for reaching a range of small molecules (100 Da) to large proteins (>300 kDa), thereby allowing measurement of metabolites, lipids, peptides and proteins [31].

In our study, the Protein ANalysis Through Evolutionary Relationships (PANTHER) analysis was performed to gain a better understanding of the functions of all differently expressed proteins. The proteins were categorized based on cellular localization, molecular function and biological process [32]. To the best of our knowledge, there was only one previously published Indian study on the proteomic analysis of OSMF and normal tissue that identified 15 upregulated genes, of which HSP-70, enolase and Lumican were also recognized in the current study [9]. However, our study, apart from samples from OSMF patients, additionally included patient samples with histologically proven OSCC along with OSMF and which were compared to normal patients, which helped in evaluating the target proteins involved in the malignant transformation of OSMF. Among the 23 upregulated proteins identified, 14-3-3ε, CA 1 and HSP 70 were found to be secretory proteins. Therefore, these protein targets were chosen with a larger number of samples for further validation using IHC.

14-3-3 proteins have been found to play a vital role at the interface between cancer, aging and age-related neurodegenerative diseases [33]. The 14-3-3 protein family consists of 2833 kDa acidic proteins that are found in all eukaryotes and are phosphorylated serine/threonine binding proteins that bind to various kinases, phosphatases, transmembrane receptors and transcription factors [34,35]. The 14-3-3 proteins generally interact with proteins involved in functions such as regulation, localization or catalysis. It is generally accepted that 14-3-3 proteins act in two ways: they either act as adapters or exhibit chaperone-like activity [36,37]. The 14-3-3 proteins regulate critical biological processes such as cell proliferation, growth and apoptosis through interaction with their partners and are thus also involved in the regulation of various tumours [38,39]. There are seven well-recognized isoforms of 14-3-3 proteins, namely 14-3-3ζ, 14-3-3σ, 14-3-3β, 14-3-3ε, 14-3-3γ, 14-3-3η and 14-3-3τ/θ in various cancers, of which 14-3-3ε has been implicated in kidney, liver, squamous cell, breast and stomach cancers [40,41]. Overexpression of 14-3-3ζ, among the various 14-3-3 proteins, has been observed in OPMD and OSCC using IHC and has therefore been suggested to play an important role in tumour development and progression [42]. The literature on 14-3-3ε, however, appears to be sparse in oral cancer and the first of its kind is reported in OSMF. Overexpression of 14-3-3ε in OSMF and OSCC shows its role in the initiation and progression of OSCC, and it may therefore be worthwhile to further investigate its association with oral tumorigenesis.

Currently, there are no studies available to correlate CA 1 in OSMF, and this study is the first of its kind showing increased expression and significant association in OSMF and OSCC when compared to normal samples. The CAs are grouped into six distinctive classes (α, β, γ, δ, τ, η), of which the α-class is detected in humans, mainly expressed in tissues with differing in pH and metabolic rate and is involved in catalyzed CO_2_ reactions of various physiological processes [43,44]. The 15 isoforms of CAs are encoded, among which 12 (CAs I–IV, CAs VA–VB, CAs VI–VII, CA IX and CAs XII–XIV) coordinate with zinc at their active site [45]. Based on cellular localization, the CA family is further subdivided into cytosolic (CAs I, II, III, VII and VIII), membrane-associated (CAs IV, IX, XII and XIV), mitochondrial (CA Va and Vb), secreted isoenzymes (CA VI) and catalytic CA isoforms (CAs X and XI). The CAs which have been implicated in cancer include CA IX, XII and XIV, among which CA IX in OSCC has been explored extensively [46,47]. Similarly, a study assessing CA IX in the plasma of OSMF and OSCC have shown increased expression [2]. The available literature on the role of CA 1 in OSCC is limited. A study done by Li et al. (2021) using IHC showed a significant correlation of CA I and CA II in OSCC [48].

HSPs are a group of proteins highly expressed during biological stress, especially inflammation, and they are thought to promote tumorigenesis by inhibiting apoptosis [49,50]. Heat shock proteins are broadly classified into five major families as the HSP 100 family (100–104 KDa), HS P90 family (82–90 KDa), HSP 70 family (68–75 KDa), HSP 60 family (58–65 KDa) and the small HSP family (15–30 KDa), based on apparent molecular weight, amino acid sequence homologies and their functional aspects [51]. Increased expression of HSP 70 was noted in leukoplakia with dysplasia and OSCC, suggested to be a marker for epithelial malignancy [52]. A study done on HSP 70 by Thubashini et al. (2011) on OSMF and OSCC using IHC showed increased expression, attributed to increased copper levels, leading to elevated oxidative stress levels in OSMF [53]. A similar significant correlation has been observed in leukoplakia and OSCC compared to normal, and hence, the authors have concluded to explore more on the therapeutic approach for OPMD and OSCC [54]. The current study also showed a high expression in OSMF and OSCC, which is concurrent with the previous study.

To the best of our knowledge, this is the first study showing 14-3-3 and CA 1 in OSMF and their interrelationships. The current study of validating the secretory markers can pave way for the assessment of 14-3-3ε and CA in saliva samples in the future. Clinically validated targets help in the development of precision therapy, thereby improving patient care [55]. Our studies show that 14-3-3ε and CA 1 are potential new targets identified for OSMF as biomarkers, along with HSP 70, which has previously been shown to be involved in OSMF as a marker of pathogenesis. Although HSP 70 has been explored in the literature, the correlation with the stages of OSMF and OSCC is sparse. The proteins validated using IHC will pave the way in the identification of novel targets for the therapy of OSMF and for preventing its malignant transformation. These three overexpressed proteins can be offered as a panel of IHC-based biomarkers for risk stratification of OSMF.

## 5. Conclusions

South East Asia has a unique increasing burden of OSMF and also a scarcity of intense research work on exclusive OSMF patients. The current study shows proteomic profiling using clinical samples, along with validation in a large series of cases. Validation studies revealed a panel of biomarkers, namely CA1, 14-3-3ε and HSP 70, to be potentially useful in identifying OSMF patients with an increased risk of oral cancer development. With currently no efficient treatment modality available, the results obtained will play a significant role in the evolution of a novel targeted therapy and in preventing malignant transformation.

## Figures and Tables

**Table 1:** List of upregulated proteins between OSMF, OSCC and normal tissues identified using MALDI-TOF.

Spot ID	Gene ID	Accession	Description	Score	Coverage	Proteins	Unique Peptides	Peptides	PSMs	AAs	MW [kDa]	calc. pI
1	MYL1	P05976	Myosin light chain 1/3, skeletal muscle isoform OS = Homo sapiens GN = MYL1 PE = 1 SV = 3-[MYL1_HUMAN]	21.57	60.31	3	4	8	12	194	21.1	5.03
2	HSPA8	P11142	Heat shock cognate 71 kDa protein OS = Homo sapiens GN = HSPA8 PE = 2 SV = 1-[P11142_HUMAN]	34.78	19.94	43	6	11	16	627	68.8	5.52
3	ENO3	P13929	Beta-enolase OS = Homo sapiens GN = ENO3 PE = 1 SV = 5-[ENOB_HUMAN]	28.92	44.47	18	4	16	19	434	47.0	7.71
4	ENO1	P06733	Alpha-enolase OS = Homo sapiens GN = ENO1 PE = 1 SV=2-[ENOA_HUMAN]	51.42	49.08	24	7	17	36	434	47.1	7.39
5	LUM	P51884	Lumican OS = Homo sapiens GN = LUM PE = 1 SV = 2-[LUM_HUMAN]	47.77	32.54	2	7	10	21	338	38.4	6.61
6	CA2	P00918	Carbonic anhydrase 2 OS = Homo sapiens GN = CA2 PE = 1 SV = 2-[CAH2_HUMAN]	121.94	55.38	3	12	12	33	260	29.2	7.40
7	CA1	P00915	Carbonic anhydrase 1 OS = Homo sapiens GN = CA1 PE=1 SV =2-[CAH1_HUMAN]	18.68	57.85	13	6	10	11	261	28.85	7.12
8	GSTP1	P09211	Glutathione S-transferase P OS = Homo sapiens GN = GSTP1 PE = 1 SV = 2-[GSTP1_HUMAN]	207.61	56.67	5	7	10	55	210	23.3	5.64
9	HBB	P68871	Hemoglobin beta (Fragment) OS = Homo sapiens GN = HBB PE = 2 SV = 1-[P68871_HUMAN]	273.10	73.33	15	0	8	171	105	11.4	6.68
10	KRT9	P35527	Keratin, type I cytoskeletal 9 OS = Homo sapiens GN = KRT9 PE = 1 SV = 3-[K1C9_HUMAN]	48.25	49.92	2	10	21	28	623	62.0	5.24
11	KRT3	P12035	Keratin, type II cytoskeletal 3 OS = Homo sapiens GN = KRT3 PE = 1 SV = 3-[K2C3_HUMAN]	22.03	23.09	8	0	15	20	628	64.4	6.48
12	KRT6C	P48668	Keratin, type II cytoskeletal 6C OS = Homo sapiens GN = KRT6C PE = 1 SV = 3-[K2C6C_HUMAN]	110.84	45.92	22	9	28	62	564	60.0	8.00
13	HBA1	P69905	Hemoglobin subunit alpha OS = Homo sapiens GN = HBA1 PE = 1 SV = 2-[HBA_HUMAN]	22.52	64.08	15	4	8	20	142	15.2	8.68
14	ALB	A0A0C4DGB6	Serum albumin OS = Homo sapiens PE = 2 SV = 1-A0A0C4DGB6_HUMAN]	159.38	50.90	13	14	31	90	609	69.0	6.20
15	ANXA2	H0YMM1	Annexin (Fragment) OS = Homo sapiens GN = ANXA2 PE = 2 SV = 1-[H0YMM1_HUMAN]	22.31	26.85	24	3	3	7	149	16.4	5.91
16	VIM	P08670	Vimentin OS = Homo sapiens GN = VIM PE = 3 SV = 1-[P08670_HUMAN]	19.27	20.88	32	5	10	19	431	49.6	5.25
17	YWHAE	P62258	14-3-3 protein epsilon OS = Homo sapiens GN = YWHAE PE = 1 SV = 1-[1433E_HUMAN]	55.66	37.25	14	5	6	16	255	29.2	4.74
18	SERPINB4	Q5K634	SCCA2/SCCA1 fusion protein isoform 1 OS = Homo sapiens PE = 2 SV = 1-[Q5K634_HUMAN]	270.44	78.72	3	0	42	91	390	44.6	6.39
19	MYL6	P60660	Myosin light polypeptide 6 OS = Homo sapiens GN = MYL6 PE = 2 SV = 1-[P60660_HUMAN]	84.22	56.55	20	7	7	26	145	16.3	4.65
20	PFN1	P07737	Profilin-1 OS = Homo sapiens GN = PFN1 PE = 1 SV = 2-[PROF1_HUMAN]	28.97	57.86	3	4	9	23	140	15.0	8.27
21	HSP90AA1	Q2VPJ6	HSP90AA1 protein (Fragment) OS = Homo sapiens GN = HSP90AA1 PE = 2 SV = 1-[Q2VPJ6_HUMAN]	49.70	34.53	30	4	17	22	585	68.3	5.19
22	KRT77	Q0IIN1	Keratin 77 OS = Homo sapiens GN = KRT77 PE = 2 SV = 1-[Q0IIN1_HUMAN]	23.03	11.42	4	1	6	8	578	61.8	5.85
23	DCD	P81605	Dermcidin OS = Homo sapiens GN = DCD PE = 1 SV = 2-[DCD_HUMAN]	9.25	10.00	1	1	1	4	110	11.3	6.54

**Table 2:** List of 23 differentially regulated proteins describing the name and regulation status obtained from Biological Variate analysis.

Spot ID	Protein	Regulation in OSMF Sample
1	Myosin light chain 1	UP
2	Heat shock 70 kDa protein	UP
3	Beta-enolase	UP
4	Alpha-enolase	UP
5	Lumican	UP
6	Carbonic anhydrase 2	UP
7	Carbonic anhydrase 1	UP
8	Glutathione S-transferase P	UP
9	Hemoglobin subunit beta	UP
10	Keratin, type I cytoskeletal 9	UP
11	Keratin, type II cytoskeletal 3	UP
12	Keratin, type II cytoskeletal 6C	UP
13	Hemoglobin subunit alpha	UP
14	Serum Albumin	UP
15	Annexin A2	UP
16	Vimentin	UP
17	14-3-3 protein epsilon	UP
18	SCCA2/SCCA1 fusion protein isoform 1	UP
19	Myosin light polypeptide 6	UP
20	Profilin-1	UP
21	HSP90AA1 protein (Fragment)	UP
22	Keratin 77	UP
23	Dermcidin	UP

**Table 3:** Clinical histopathological features in CA 1-positive and -negative groups of OSMF, OSCC and normal patients.

Criteria	Total (*n* = 125)	CA 1-Negative (*n* = 37)	CA 1-Positive (*n* = 88)
**Age**			
<42 yrs	60	21 (35%)	39 (65%)
>42 yrs	65	16 (24.6%)	49 (75.4%)
**Gender**			
Male	93	29 (31.2%)	64 (68.8%)
Female	32	8 (25%)	24 (75%)
**OSMF Clinical Stage (*n* = 72)**			
Stage I	16	4 (25%)	12 (75%)
Stage II	28	10 (35.7%)	18 (64.3%)
Stage III	26	2 (7.7%)	24 (92.3%)
Stage IV	2	0 (0%)	2 (100%)
**Habits (OSMF *n* = 72)**			
Pan	33	9 (27.3%)	24 (72.7%)
Betel Nut	20	5 (25%)	15 (75%)
Maava	13	2 (15.4%)	11 (84.6%)
Gutka	6	0 (0%)	6 (100%)
**Vascularity (OSMF *n* = 72)**			
Normal	10	2 (20%)	8 (80%)
Reduced	40	11 (27.5%)	29 (72.5%)
Increased	20	3 (15%)	17 (85%)
Enlarged and Increased	2	0 (0%)	2 (100%)
**OSCC Histological Stage (*n* = 40)**			
Well-differentiated OSCC	28	8 (28.6%)	20 (71.4%)
Moderately differentiated OSCC	10	2 (20%)	8 (80%)
Poorly differentiated OSCC	2	0 (0%)	2 (100%)
**OSCC Clinical Stage (*n* = 40)**			
Stage I	8	2 (25%)	6 (75%)
Stage II	12	3 (25%)	9 (75%)
Stage III	6	1 (16.7%)	5 (83.3%)
Stage IV	14	4 (28.6%)	10 (71.4%)
**Inflammation**			
No	6	3 (50%)	3 (50%)
Mild	32	11 (34.4%)	21 (65.6%)
Moderate	55	14 (25.5%)	41 (74.5%)
Severe	32	9 (28.1%)	23 (71.9%)
**Fibrosis (OSMF *n* = 72)**			
Mild	18	5 (27.8%)	13 (72.2%)
Moderate	28	8 (28.6%)	20 (71.4%)
Severe	26	3 (11.5%)	23 (88.5%)
**Significant Factors**			
**Diagnosis**			
Normal	13	11 (84.6%)	2 (15.4%)
OSMF	72	16 (22.2%)	56 (77.8%)
OSCC	40	10 (25%)	30 (75%)
*p* value = 0.000; χ2 = 21.169			
**Epithelial Nature**			
Normal	30	16 (53.3%)	14 (46.7%)
Atrophic	49	10 (20.4%)	39 (79.6%)
Atrophic+mild dysplasia	1	0 (0%)	1 (100%)
Atrophic+moderate dysplasia	5	1 (20%)	4 (80%)
OSCC	40	10 (25%)	30 (75%)
*p* value = 0.025; χ2 = 11.144			

**Table 4:** Clinical histopathological features in 14-3-3ε-positive and -negative groups of OSMF, OSCC and normal patients.

Criteria	Total (*n* = 130)	14-3-3ε-Negative (*n* = 24)	14-3-3ε-Positive (*n* = 106)
**Gender**			
Female	32	4 (12.5)	28 (87.5)
Male	98	20 (20.4)	78 (79.6)
**OSMF Clinical Stage (*n* = 77)**			
Stage I	19	5 (26.3%)	14 (73.7%)
Stage II	29	4 (13.8%)	25 (86.2%)
Stage III	26	1 (3.8%)	25 (96.2%)
Stage IV	3	1 (33.3%)	2 (66.7%)
**Habits (OSMF *n* = 77)**			
Pan	36	7 (19.4%)	29 (80.6%)
Maava	13	3 (23.1%)	10 (76.9%)
Gutka	6	0 (0%)	6 (100%)
Betel nut	22	1 (4.5%	21 (95.5%)
**Fibrosis (OSMF *n* = 77)**			
Mild	20	3 (25%)	15 (75%)
Moderate	29	3 (10.3%)	26 (89.7%)
Severe	28	3 (10.7%)	25 (89.3%)
**Vascularity (OSMF *n* = 77)**			
Normal	12	4 (33.3%)	8 (66.6%)
Reduced	43	6 (14%)	37 (86%)
Increased	20	1 (5%)	19 (95%)
Enlarged and increased	02	0 (0%)	2 (100%)
**OSCC Histological Stage (*n* = 40)**			
Well-differentiated OSCC	28	2 (7.1%)	26 (92.9%)
Moderately differentiated OSCC	10	1 (10%)	9 (90%)
Poorly differentiated OSCC	2	0 (0%)	2 (100%)
**OSCC Clinical Stage (*n* = 40)**			
Stage I	8	0 (0%)	8 (100%)
Stage II	12	0 (0%)	12 (100%)
Stage III	6	0 (0%)	6 (100%)
Stage IV	14	3 (21.4%)	11 (78.6%)
**Significant**			
**Age**			
<43 yrs	64	17 (26.6%)	47 (73.4%)
>43 yrs	66	7 (10.6%)	59 (89.4%)
*p* value = 0.01; χ2 = 5.496			
**Diagnosis**			
Normal	13	10 (76.9%)	3 (23.1%)
OSMF	77	11 (14.3%)	66 (85.7%)
OSCC	40	3 (7.5%)	37 (92.5%)
*p* value = 0.000; χ2 = 33.600			
**Epithelial Nature**			
Normal	30	12 (40%)	18 (60%)
Atrophic + mild dysplasia	1	0 (0%)	1 (100%)
Atrophic+ moderate dysplasia	5	1 (20%)	4 (80%)
Atrophic	54	8 (14.8%)	46 (85.2%)
Malignant	40	3 (7.5%)	37 (92.5%)
*p* value = 0.010; χ2 = 13.149			
**Inflammation**			
No	6	1 (16.7%)	5 (83.3%)
Mild	32	11 (34.4%)	21 (65.6%)
Moderate	60	6 (10%)	54 (90%)
Severe	32	6 (18.8%)	26 (81.2%)
*p* value = 0.041; χ2 = 8.252			

**Table 5:** Clinical histopathological features in HSP 70-positive and -negative groups of OSMF, OSCC and normal patients.

Criteria	Total (*n* = 117)	HSP 70-Negative (*n* = 36)	HSP 70-Positive (*n* = 81)
**Gender**			
Male	85	27 (31.8%)	58 (68.2%)
Female	32	9 (28.1%)	23 (71.9%)
**OSMF Clinical Stage (*n* = 47)**			
Stage I	10	3 (30%)	7 (70%)
Stage II	18	9 (50%)	9 (50%)
Stage III	16	2 (12.5%)	14 (87.5%)
Stage IV	3	1 (33.3%)	2 (66.7%)
**Habits (OSMF *n* = 47)**			
Pan	28	10 (35.7%)	18 (64.3%)
Betel Nut	7	2 (28.6%)	5 (71.4%)
Maava	10	2 (20%)	8 (80%)
Gutka	2	1 (50%)	1 (50%)
**Inflammation**			
Mild	44	19 (43.2%)	25 (56.8%)
Moderate	49	12 (24.5%)	37 (75.5%)
Severe	24	5 (20.8%)	19 (79.2%)
**Fibrosis (OSMF *n* = 47)**			
Mild	10	3 (30%)	7 (70%)
Moderate	18	5 (27.8%)	13 (72.2%)
Severe	19	7 (36.8%)	12 (63.2%)
**Vascularity (OSMF *n* = 47)**			
Normal	7	3 (42.9%)	4 (57.1%)
Reduced	23	8 (34.8%)	15 (65.2%)
Increased	17	4 (23.5%)	13 (76.5%)
**OSCC Histological Stage (*n* = 53)**			
Well-differentiated OSCC	25	6 (24%)	19 (76%)
Moderately differentiated OSCC	24	5 (20.8%)	19 (79.2%)
Poorly differentiated OSCC	4	0 (0%)	4 (100%)
**OSCC Clinical Stage (*n* = 53)**			
Stage I	9	1 (11.1%)	8 (88.9%)
Stage II	17	1 (5.9%)	16 (94.1%)
Stage III	13	5 (38.5%)	8 (61.5%)
Stage IV	14	4 (28.6%)	10 (71.4%)
**Significant**			
**Age**			
<47 yrs	57	24 (42.1%)	33 (57.9%)
>47 yrs	60	12 (20%)	48 (80%)
*p* value = 0.016; χ2 = 6.705			
**Diagnosis**			
Normal	17	10 (58.8%)	7 (41.2%)
OSMF	47	15 (31.9%)	32 (68.1%)
OSCC	53	11 (20.8%)	42 (79.2%)
*p* value = 0.012; χ2 = 8.805			
**Epithelial Nature**			
Normal	9	5 (55.6%)	4 (44.4%)
Hypertrophic	16	8 (50%)	8 (50%)
Atrophic	36	12 (33.3%)	24 (66.7%)
Dysplasia	03	0 (0%)	3 (100%)
OSCC	53	11 (20.8%)	42 (79.2%)
*p* value = 0.05; χ2 = 9.313			

## Data Availability

The study did not report any additional data.

## Supplementary Materials

The following supporting information can be downloaded at: https://www.mdpi.com/article/10.3390/jpm12020208/s1, Figure S1: Functional classification of 23 differentially regulated proteins and biological network analysis using PANTHER gene ontology database, were functionally categorized as based on: (A) Molecular Function, (B) Biological Process, (C) Cellular Component, Figure S2: (A) Protein classification, (B) pathway analysis using PANTHER database for 23 differentially upregulated proteins, Table S1: Pathway analysis data of carbonic anhydrase 1 (CA 1), Table S2: Pathway analysis data of 14-3-3ε, Table S3: Pathway analysis data of HSP 70.
