# Supplementary material for: Integrated Proteomics Based on 2D Gel Electrophoresis and Mass Spectrometry with Validations: Identification of a Biomarker Compendium for Oral Submucous Fibrosis—An Indian Study
Source: J Pers Med. 2022 Feb 3;12(2):208. doi: 10.3390/jpm12020208 (PMC8878868; doi:10.3390/jpm12020208)
Supplement: Supplementary file 1 [file jpm-12-00208-s001.zip › jpm-1569101-supplementary.pdf]

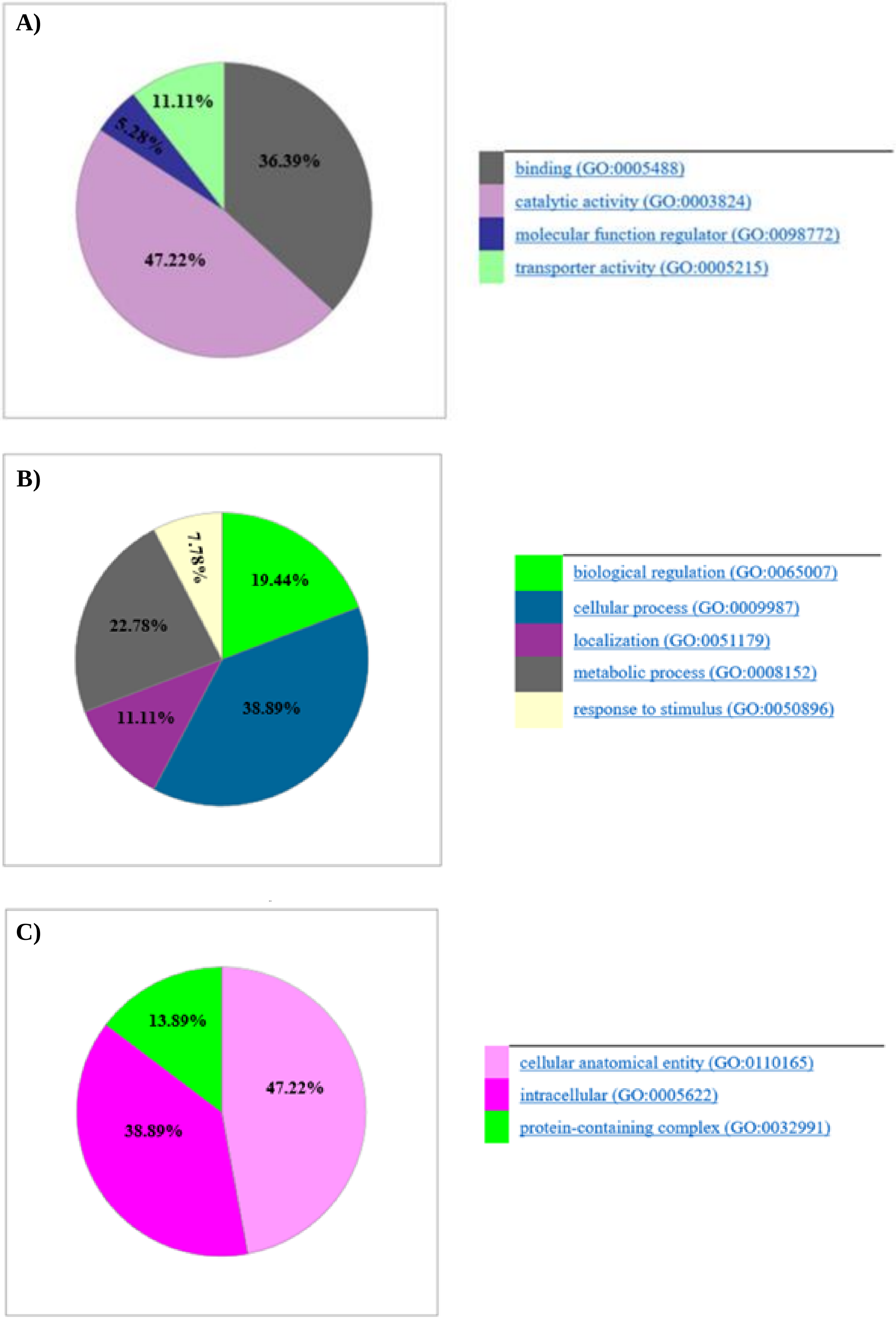

Figure S1. Functional classification of 23 differentially regulated proteins and biological network analysis using PANTHER gene ontology database, were functionally categorized as based on: **A)** Molecular Function, **B)** Biological Process, **C)** Cellular Component

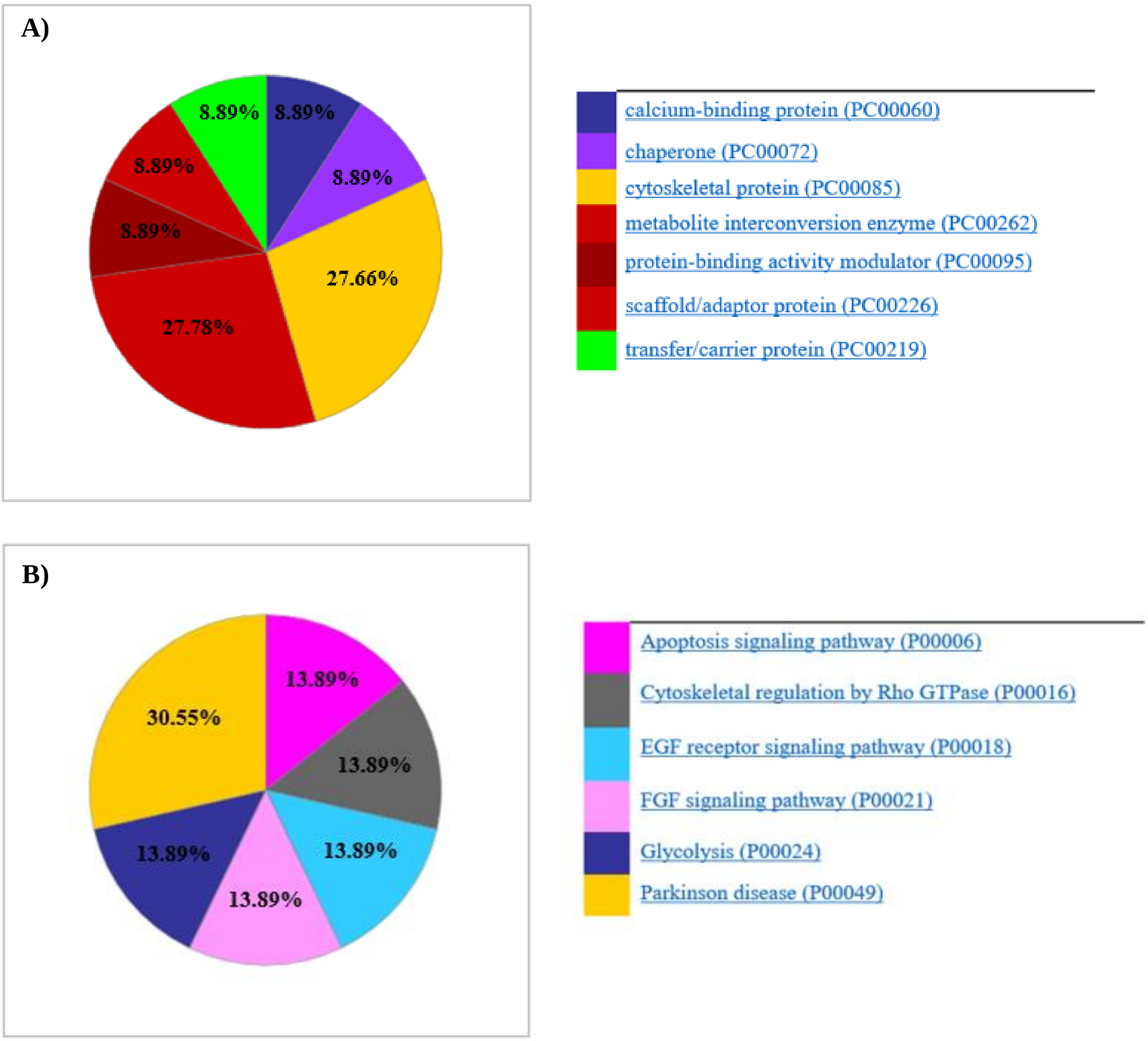

Figure S2. A) Protein classification and **B)** pathway analysis using PANTHER database for 23 differentially upregulated proteins

Table S1. Pathway analysis data of carbonic anhydrase 1 (CA 1)

Qualifier	Name	GO Term	%	Count
Part of	cytosol	Cellular Component	20	5
Enables	carbonate dehydratase activity	Molecular Function	16	4
Part of	cytoplasm	Cellular Component	12	3
Involved In	bicarbonate transport	Biological Process	12	3
Enables	protein binding	Molecular Function	8	2
Enables	arylesterase activity	Molecular Function	4	1
Involved In	one-carbon metabolic process	Biological Process	4	1
Enables	zinc ion binding	Molecular Function	4	1
Enables	lyase activity	Molecular Function	4	1
Enables	hydro-lyase activity	Molecular Function	4	1
Involved In	interleukin-12-mediated signaling pathway	Biological Process	4	1
Enables	metal ion binding	Molecular Function	4	1
Part of	extracellular exosome	Cellular Component	4	1

Table S2. Pathway analysis data of 14-3-3.

Qualifier	Name	GO Term	%	Count
Enables	protein binding	Molecular Function	41.49	78
Part of	cytosol	Cellular Component	19.15	36
Part of	cytoplasm	Cellular Component	2.13	4
Part of	extracellular exosome	Cellular Component	2.13	4
Part of	nucleus	Cellular Component	1.60	3
Enables	ion channel binding	Molecular Function	1.60	3
Enables	enzyme binding	Molecular Function	1.06	2
Enables	protein domain specific binding	Molecular Function	1.06	2
Enables	identical protein binding	Molecular Function	1.06	2
Enables	histone deacetylase binding	Molecular Function	1.06	2
Involved In	G2/M transition of mitotic cell cycle	Biological Process	0.53	1
Involved In	MAPK cascade	Biological Process	0.53	1
Involved In	neuron migration	Biological Process	0.53	1
Involved In	regulation of heart rate by hormone	Biological Process	0.53	1
Enables	RNA binding	Molecular Function	0.53	1
Enables	calcium channel regulator activity	Molecular Function	0.53	1
Part of	cell	Cellular Component	0.53	1
Part of	mitochondrion	Cellular Component	0.53	1
Part of	kinesin complex	Cellular Component	0.53	1
Part of	plasma membrane	Cellular Component	0.53	1
Part of	focal adhesion	Cellular Component	0.53	1
Involved In	protein targeting	Biological Process	0.53	1
Involved In	regulation of G2/M transition of mitotic cell cycle	Biological Process	0.53	1
Enables	potassium channel regulator activity	Molecular Function	0.53	1
Part of	membrane	Cellular Component	0.53	1
Involved In	viral process	Biological Process	0.53	1

Involved In	substantia nigra development	Biological Process	0.53	1
Involved In	hippocampus development	Biological Process	0.53	1
Involved In	cerebral cortex development	Biological Process	0.53	1
Enables	MHC class II protein complex binding	Molecular Function	0.53	1
Part of	axon	Cellular Component	0.53	1
Enables	ubiquitin protein ligase binding	Molecular Function	0.53	1

Table S3. Pathway analysis data of HSP 70

Name	Qualifier	GO Term	%	Count
ATP binding	Enables	Molecular Function	28.57	2
nucleotide binding	Enables	Molecular Function	14.29	1
protein folding	Involved In	Biological Process	14.29	1
ATPase	Enables	Molecular Function	14.29	1
melanosome	Part of	Cellular Component	14.29	1
unfolded protein binding	Enables	Molecular Function	14.29	1
